# Research on paths of opportunistic behavior avoidance and performance improvement in food supply chain from the perspective of social control

**DOI:** 10.3389/fpsyg.2022.1101543

**Published:** 2023-01-12

**Authors:** Tu Lyu, Yulin Guo, Qixiang Geng

**Affiliations:** School of Business, Qingdao University, Qingdao, China

**Keywords:** social control, information sharing, opportunistic behavior, supply chain performance, joint planning, joint problem solving

## Abstract

It is essential to avoid opportunistic behaviors of food supply chain members to guarantee food safety and sustainable supply. This research adopted the perspective of supply chain membership governance to discuss the critical mechanisms of opportunistic behavior avoidance and performance improvement in the food supply chain. Two information-sharing mechanisms (information sharing with customers and information sharing with suppliers) were used as mediating variables to explore the mechanisms of how social control, information sharing, and opportunistic behavior worked on supply chain performance. Furthermore, an online questionnaire survey was conducted to collect 210 data samples from the food manufacturing industry in China, and the structural equation model method was applied to test the research hypotheses. According to the empirical research findings, social control can directly reduce opportunistic behaviors of supply chain members and reduce such behaviors indirectly *via* the mediating factor of information sharing; social control affects the supply chain performance *via* the mediating factors of information sharing and opportunistic behavior, instead of directly improving supply chain performance. Two information sharing mechanisms vary in their mechanism of influence. Information sharing with customers reduces opportunistic behaviors, but does not directly improve supply chain performance. Information sharing with suppliers enhances supply chain performance and reduces opportunistic behaviors. This research offers theoretical and practical suggestions for performance improvement and opportunistic behavior avoidance to promote food supply chain management.

## 1. Introduction

Food safety and sustainable supply have always been a hot topic of great concern to the public both in China and foreign countries, because it is a matter of physical health and social stability. Especially in the context of regular prevention and control of COVID-19, the food supply chain has undertaken severe financial and survival pressures because the human capital flow was restricted, consumer demands have changed, food production facilities and factories have closed, and food trade has been limited (Aday and Aday, 2020; Deconinck et al., 2021; Luckstead et al., 2021). This would easily trigger the occurrence of opportunistic behaviors, such as selling shoddy goods, cheating on the quantity of the goods, and delaying the food supply, which badly hinders the normal running of the food supply chain (Huo et al., 2019; Yadav et al., 2021) and even seriously affects food safety and supply (Kumar and Singh, 2022).

Opportunism is a dishonest breach of business obligations and “self-interest seeking with guile” (Williamson, 1998). Dependence (Gorton et al., 2015), uncertainty (Dahlstrom et al., 2009), justice (Huo et al., 2016b), legal contracts (Son et al., 2021), relational norms (Paswan et al., 2017; Son et al., 2021), buyer coercive and non-coercive powers (Wang et al., 2015) are factors that either increase or decrease the likelihood of opportunism. Scholars have conducted studies on achieving the recovery and stable operation of the food supply chain to reduce the food supply problems arising from chain disruption and mitigate food safety risks (Barman et al., 2021). It is especially worth noting that scholars have made great efforts to reduce the opportunistic behavior of food supply chain member enterprises. For example, Tran et al. (2021) focused on food suppliers’ opportunistic behaviors, such as selling products to others, mixing products, cheating on the supply quantity, cheating on the product quality, refusing to use input provided, and wrongly using money, and presented suggestions for reducing the opportunistic behaviors. Liu (2018) argued that relationship-specific investments could reduce members’ distrust and opportunistic behavior in the food supply chain. Nakandala et al. (2020) pointed out that strengthening mutual trust is an important measure to reduce opportunism in the food supply chain to build shared power and fairness. Xu et al. (2020) maintained that blockchain technology could create a food traceability supply chain to eliminate potential food safety hazards. Similarly, Fu et al. (2020) believed that emerging information technologies such as blockchain and the Internet of things could contribute to easing the constraints of information asymmetry in the food supply chain and reducing opportunistic behaviors.

We assume that the social control mechanism can offer new perspectives for reducing opportunistic behaviors in the supply chain context. Building social control mechanisms to enhance supply chain performance has been confirmed to be a feasible way to maintain favorable relationships between supply chain members (Teller et al., 2016). Different from to use of contractual governance (Jia et al., 2020) or formal control (Li et al., 2010) to reduce opportunism in a supply chain context, which may pay a price of high costs, social control, based on the relationship management of supply chain members, strengthens the mutual trust of upstream and downstream enterprises to seek joint planning and problem solving and improve information exchanges to lower the uncertainty and inventory level (Acharyulu and Shekbar, 2012), thus eventually bringing the likelihood of reducing opportunism. In the supply chain, social control is an effective means to minimize opportunism and improve supply chain performance, which helps supply chain member enterprises to choose favorable partners, lower transaction costs, strengthen the core competitiveness, and thus improve the overall supply chain performance (Cheng, 2021). Meanwhile, Wu et al. (2014) pointed out that social control has the high-level strategy of reducing the “free-riding” behavior of enterprises to achieve mutual understanding and knowledge sharing between upstream and downstream supply chain enterprises, thus enhancing supply chain performance. Li et al. (2010) argued that social control spurs supply chain partners to increase investment in mutual benefit, improving supply chain performance. Falk and Kosfeld (2006) found that trust in social control would raise the hidden cost of opportunistic behaviors among supply chain partners, thus contributing to reducing opportunistic behaviors of enterprises. Beyond that, social control can increase the likelihood of upstream and downstream enterprises working jointly to fulfill the same goal, thus further facilitating enterprise cooperation. Cheng (2021) concluded that profits increase along with the increase in the degree of social control, and social control can increase the safety and credibility of transactions, thereby enhancing the overall performance of the supply chain. Food supply chain enterprises can build social control mechanisms to boost cooperation among member enterprises, lower transaction costs, strengthen information and knowledge sharing, adopt flexible and effective measures to deal with changes in the market environment, and respond quickly to uncertainty, to reduce opportunistic behaviors of supply chain members and enhance the overall performance of the supply chain. Therefore, this research has the primary objective of discussing whether social control mechanisms can restrain food supply chain enterprises and thus achieve the effect of reducing opportunism and enhancing the overall performance of the supply chain.

In the context of regular COVID-19 prevention and control, market information changes rapidly and bears great uncertainty. Food manufacturing enterprises must have quick access to helpful information in the business environment and share it with partners timely (Barman et al., 2021). Therefore, it is highly urgent to build an enterprise information-sharing architecture. Information sharing is needed for the food supply chain. Information sharing can help enterprises to rationally allocate their supply chain resources and cut losses and waste incurred by dynamic environmental changes. Information sharing can also optimize the supply chain structure and propel overall goal fulfillment (Zhou and Benton, 2007). Information sharing among food supply chain enterprises can enhance the overall quality of the supply chain (Ding et al., 2014; Christensen et al., 2021; Liu et al., 2021). Information sharing calls for effective coordination and communication among organizations to cope with the increasingly-complex market environment, which also reveals the core competitive advantage of the supply chain (Shepherd and Günter, 2010). Upstream and downstream manufacturing enterprises should build an intimate and mutually-beneficial relationship, because such a member relationship-based social control mode can help enterprises to exchange information and access external critical information and technology, thereby reducing opportunistic behaviors (You et al., 2018). Especially for food manufacturing enterprises, timely information exchange with supply chain partners can be conducive to improving production plans, stimulating production vitality, improving the food quality and supply capacity, reducing speculative activities caused by information asymmetry between enterprises, and enhancing the efficiency and effectiveness of the supply chain. Accordingly, social control, information sharing, opportunistic behavior, and supply chain performance are interrelated. Our second objective of this research is to investigate the critical effect of information sharing on the relationship between social control, opportunistic behavior, and supply chain management performance.

In summary, this research investigates how social control influences the opportunistic behavior and supply chain performance of enterprises in the food supply chain and attempts to discuss the mediating role of information sharing in the mechanism. Our study contributes to the literature in the following respects. Firstly, from the perspective of social control and information sharing, this study revealed the critical antecedents of reducing opportunistic behavior in food supply chain firms. Secondly, our research contributes to the research by constructing and discovering the mediating mechanism of social control mechanism to improve the performance of the food supply chain; Thirdly, the research further reveals the differences between the two types of information sharing in the process of improving the performance of food supply chain. Our study also provides theoretical references for the performance improvement of the food supply chain from the perspective of social control.

## 2. Literature review and research hypotheses

Organizational information processing theory (OIPT) suggests that an organization is an open information processing system to collect, analyze, and use information effectively and efficiently, especially when executing complex tasks with extensive levels of uncertainty and interdependence (Srinivasan and Swink, 2018). A better match between the needs and capabilities of information processing can ensure the healthy operation of the enterprise (Li et al., 2019), and such an information processing mechanism is needed to cope with uncertainties and manage unforeseen events that threaten the normal operations of business processes (Wong, C. W. Y. et al., 2020). In addition to strengthening internal information processing, it is an essential way for enterprises to conduct information processing to build communication channels by establishing contacts with other organizations, such as direct contacts between different enterprises to facilitate processes of joint decision making (Galbraith, 1974). Information exchange with suppliers and customers to obtain current and valuable information can improve the visibility of information and increase the effectiveness of enterprise decision-making (Srinivasan and Swink, 2018; Han et al., 2021). Studies suggested that information sharing is an important strategy to enhance information processing capabilities (Wong, C. Y. et al., 2020); therefore, based on the OIPT, this study complied with the research logic of “information acquisition—information processing—performance production” to develop our research model to capture the antecedents and consequences of information sharing.

The food supply chain is witnessing a more fast-changing and chaotic market environment because of the information economy, economic globalization, and the pandemic (Aday and Aday, 2020; Rizou et al., 2020). The difficulty in predicting the market poses more challenges for the stable development of the food supply chain. Enterprises effectively share supplier and customer information with upstream and downstream enterprises can achieve better exchanges and get excessive heterogeneous resources (Tran et al., 2021; Lyu et al., 2023). The utilization of information sharing resources can bring a unique competitive advantage to real-time information exchanges between the enterprise and its upstream enterprises (suppliers) and downstream enterprises (distributors). With this competitive advantage, the supply chain can become more special, because resource endowments from the competitive advantage create a more incredible difficulty for other manufacturing supply chains to imitate (Battistelli et al., 2019). Therefore, information sharing is a necessary information processing mechanism for food supply chain enterprises no matter from the perspective of OIPT theory or the realistic demand of the above analysis.

Prior studies of food supply chains confirmed that a good supply chain relationship quality is a crucial precursor for any stable exchange relationship that ensures relationship continuity and high performance (Odongo et al., 2016; Mesic et al., 2018). Building a good relationship with supply chain members makes it feasible to provide a way for enterprises to access and utilize resources in the supply chain. This way, enterprises can better cooperate with their supply chain partners and develop competitive advantages (Wu et al., 2022). From the perspective of relationship maintenance, food supply chain members can adopt social control measures such as trust accumulation and joint efforts to promote the expansion or stabilization of relational capital (Badraoui et al., 2020), and stimulate knowledge interaction and information sharing among supply chain members (Dawuti, 2014). Accordingly, we connected social control and information sharing to deconstruct the “information acquisition—information processing” relationship.

**Figure 1:**
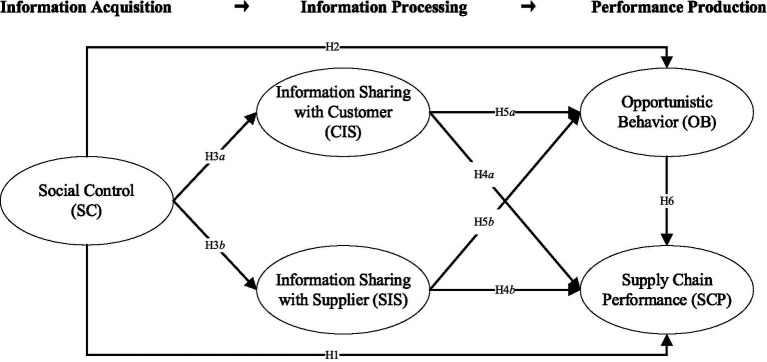
Research model.

Performance production in this study includes avoiding or reducing opportunistic behaviors and increasing supply chain performance. Rapid market changes will bring more uncertainty and trigger opportunistic behaviors of food supply chain enterprises (Liu, 2018; Xu et al., 2020). Therefore, food supply chain enterprises must work together to take measures to avoid uncertainty. This research argues that social control and information sharing can be introduced to weaken the influence of environmental changes on opportunistic behaviors. Social control will spur enterprises to constantly detect problems, integrate resources, and allocate resources to reduce opportunistic behavior, thus responding to the market and gratifying customer needs (Wu et al., 2014); Information sharing can keep the supply chain balanced and stable and reduce uncertainty and opportunism (You et al., 2018). Besides, previous studies confirmed that social control and information sharing are drivers to promote supply chain performance in various research scenarios (Anin et al., 2016; Shibin et al., 2020). Therefore, food manufacturing enterprises should implement long-term social control. In addition, the enterprises should continue to strengthen the information sharing between suppliers and customers, thus satisfying the market demand, maintaining the stability and development of the supply chain, and guaranteeing food quality and sustained supply. In summary, the research model shown in Figure 1 reveals the relationships and influence mechanisms of social control, information sharing, opportunistic behavior, and supply chain performance.

Social control mechanism consists of trust, joint planning, joint problem solving, and other core contents (Huang et al., 2014), which have been implemented in food supply chain management (Dania et al., 2018). Trust can forge a basis for building cooperative relationships among food supply chain members (Dania et al., 2018). It is helpful to reduce the risk associated with opportunistic behavior (Wu et al., 2014) and significantly increase supply chain operational performance (Ramirez et al., 2021). Joint planning helps the upstream and downstream enterprises of the food supply chain to develop a shared development strategy, development direction, and long-term goals, and integrate limited resources to maintain sustainable behavior (Dania et al., 2018). Joint problem solving encourages upstream and downstream enterprises to work together to adjust uncertainty and conflicts, and enhances the opportunities for communication between the upstream and downstream enterprises to reduce opportunistic behaviors (Cai et al., 2009; Rhee et al., 2014). Thus, the following hypotheses are developed:

*H1*: Social control positively influences the food supply chain performance.

*H2*: Social control negatively influences opportunistic behavior.

Distinguishing the information shared by upstream and downstream enterprises can achieve more favorable sharing effects (Alzoubi and Yanamandra, 2020). Guan et al. (2020) found that information sharing between manufacturing enterprises and suppliers can boost the profits of upstream and downstream enterprises, bring scale economies and save operating costs. Prajogo and Olhager (2012) argued that information sharing between manufacturing enterprises and customers could facilitate enterprises to get market information, such as the demand of downstream customers, thus exploiting the customer value and altering product research and development strategies. According to prior studies, this research classifies information sharing into two categories: information sharing with customers and suppliers.

Social control enables enterprises in the supply chain to access critical information from multiple perspectives and channels of various types (Abdullah and Musa, 2014). In particular, food supply chain members have begun to share information about food processing, procurement, inventory, and marketing (Kumar and Singh, 2022). Tajvidi et al. (2020) concluded that social control could provide a strong guarantee for information sharing and lower the risk of necessary information disclosure so enterprises can focus on value co-creation. Wu et al. (2014) found that by establishing a favorable social control mechanism, core enterprises can build a long-term cooperative relationship with upstream and downstream enterprises and raise their information-sharing willingness, thus enhancing the supply chain performance. Information sharing with suppliers and consumers can strengthen the responsiveness of both suppliers and manufacturers (Han et al., 2021), improve the traceability of food sources, strengthen the innovation and flexibility of the supply chain, lower the cost of food production and processing (Reklitis et al., 2021), and safeguard and coordinate the interest of supply chain members (Dania et al., 2018). We thus provided the following hypotheses:

*H3*: Social control positively influences information sharing (a. information sharing with customers; b. information sharing with suppliers).

The efficient implementation of information sharing can reduce the uncertainty of the supply chain in business operations and help manufacturing enterprises to acquire critical resources, thereby enhancing the dynamic capability and performance of the supply chain (Shan et al., 2020; de Lima et al., 2022; Lyu et al., 2022), and reducing the occurrence of opportunistic behaviors (Huo et al., 2021). In our research context, information sharing among food supply chain members can effectively enhance supply chain performance (Sener et al., 2019; Sundram et al., 2020). Strengthening information exchanges with customers and suppliers can facilitate the timely feedback of end customers’ demands to suppliers and customized production in specific demand scenarios. Especially in the pandemic context, such customized food production and supply can reduce the costs of enterprises, improve the suitability of products to the market, guarantee the sales and market value of food, and ease the burden on manufacturing enterprises. Beyond that, such a full supply chain-based information exchange mode can avoid opportunistic behaviors triggered by information asymmetry and enhance the cooperation efficiency of the supply chain while regulating enterprise behavior. Based on the above analysis, the following hypotheses are presented:

*H4*: Information sharing (a. information sharing with customers; b. information sharing with suppliers) positively influences the food supply chain performance.

*H5*: Information sharing (a. information sharing with customers; b. information sharing with suppliers) negatively influences opportunistic behavior.

Given the uncertainty of information exchanges, the supply chain is prone to opportunistic behaviors, which infringe on the stable and long-term cooperative relationship of manufacturing enterprises and incur risks and profit losses (Xu et al., 2020) and have specific manifestations in withholding and distorting information and refusing to fulfill commitments and obligations (Zhang and Chen, 2013). As suppliers and distributors have different rights and positions, enterprises in a weak position should invest more to communicate and build relationships, to weaken enterprises’ motivation to engage in opportunistic behaviors (You et al., 2018). For food supply chain enterprises, such opportunistic behaviors of partners adversely affect the performance of food manufacturing enterprises, and, worse still, may trigger a series of potential problems such as food safety and belated supply (Tran et al., 2021). Based on the above analysis, this research develops the following hypothesis:

*H6*: Opportunistic behavior negatively influences supply chain performance.

## 3. Research methodology

### 3.1. Measurement development

To guarantee that the research data can fully reflect reality and accord with the requirements of reliability and validity, this research used the proven scales in the existing literature as a reference for design and measurement. In particular, the scales used in the Chinese scenario were adjusted to accord with the food supply chain scenario in this research. Based on Claro et al. (2003) and Cai et al. (2009), social control was measured from three dimensions: trust, joint planning, and joint problem solving. Scales of trust and joint planning were designed to have three items, respectively, and the scale of joint planning had four items. Adapted from Flynn et al. (2010) and Prajogo and Olhager (2012), information sharing was measured from two dimensions: information sharing with suppliers and information sharing with customers. The scale of information sharing with customers had three items, while the scale of information sharing with suppliers had four items. Meanwhile, five items were designed based on the research of Huo et al. (2017) and Prajogo and Olhager (2012) to develop supply chain performance scales. Using the research of Luo et al. (2015) and Huo et al. (2016a) for reference, this research designed four items to measure opportunistic behaviors. The enterprise age, size, and property were also used as control variables. The questionnaire adopted a 5-point Likert scale to describe the degree of agreement with statements in the question item (indicating 1 = Strongly Disagree, 2 = Disagree, 3 = Neutral, 4 = Agree, and 5 = Strongly Agree).

### 3.2. Pilot study

We followed Rasool et al. (2021) to conduct a two-stage pilot test. Firstly, to ensure the research validity, this research invited ten supply chain management personnel as respondents to engage in a pre-survey. On this basis, efforts were made to carefully consider and repeatedly revise the arcane and ambiguous words in the questionnaire and the logical relationship and positional relationship between the sentences in the questionnaire. Second, this study invited 67 MBA students to conduct a small sample pilot test. All these students are department heads or higher-level managers in food processing and manufacturing enterprises. We tested the reliability and validity of the sample data. Results found that the Cronbach’s ɑ coefficient and composite reliability (CR) of each variable are all above 0.7, and average variances extracted (AVE) and factor loading were all above 0.5, indicating that the scale involved in this study could measure the relevant variables more accurately.

### 3.3. Data collection

This research chose the online survey approach for data collection to avoid the limitations of financial resources, labor power, time, and other objective factors and facilitate the questionnaire distribution and collection. Middle-senior supply chain managers from food processing and manufacturing enterprises were selected as the respondents, because they were familiar with the internal and external processes of enterprise running, including procurement, manufacturing, warehousing, transportation, and other links. In the final survey, a total of 411 samples were collected. However, in the questionnaire filtering, 101 samples were found to have a short questionnaire-filling time (much faster than the expected time), give the same option or just two options for all questionnaire items, or choose obviously-conflicting answers. After the invalid questionnaires were deleted, 310 valid samples were used for future analysis. Thus, the recovery rate of valid questionnaires was 73.0%. The basic information on the enterprise samples is shown in Table 1.

**Table 1:** Basic information of questionnaire respondents (*N* = 310).

Variable	Category	Number	%
Gender	Male	168	54.2
	Female	142	45.8
Age (years)	20–30	69	22.3
	31–40	110	35.5
	41–50	88	28.4
	More than 50	43	13.9
Education	High school and below	20	6.5
	Undergraduate and junior college	211	68.1
	Master and PhD	76	24.5
	Others	3	0.9
Position	Senior-manager	129	41.6
	Middle-manager	181	58.4
Firm Age	Less than 1 year	5	1.6
	1–3 years	68	21.9
	3–5 years	184	59.4
	More than 5 years	53	17.1
Firm Size	Less than 50 people	38	12.3
	51–100 people	146	47.1
	101–300 people	121	39.0
	More than 300 people	5	1.6
Industry	Leisure food processing	88	28.4
	Fruit and vegetable processing	14	4.5
	Fermented products	32	10.3
	Aquatic product	67	21.6
	Cereals-oil food	14	4.5
	Egg food	34	11.0
	Drinks	33	10.6
	Dairy products	14	4.5
	Food additive industry	2	0.6
	Animal by-products	3	1.0
	Candy	7	2.3
	Dietary supplement	2	0.6

## 4. Data analysis and results

### 4.1. Common method bias and multicollinearity

Since the primary method of data collection utilized by the research questionnaire is self-reporting, the likelihood of there being a common method variance (CMV) has been increased, therefore, we conducted the Harman’s single-factor analysis approach, and the results showed that CMV is not an issue because only one single factor was extracted to explain 31.5% of the variance in the endogenous variables (less than 50%; Hair et al., 2016). We also tested the VIF values to test whether multi-collinearity exists. Results suggested that all the VIF values of variables are between 1.575 and 1.891, far less than 3.3 (Diamantopoulos and Siguaw, 2006), indicating that the multi-collinearity is not an issue needs to be considered in this study.

### 4.2. Measurement validation

We used the SmartPLS 4 to test the reliability and validity of the data. As shown in Table 2, the CA scores were higher than 0.6, the CR scores were higher than 0.7, and the AVE values were higher than 0.5, which met the requirements specified by Bagozzi and Yi (1988). As shown in Table 3, the factor loadings of all items were higher than the threshold value of 0.7 specified by Chin (1998). Thus, the data had good reliability and convergent validity.

**Table 2:** Reliability and validity testing results.

Constructs	CA	CR	AVE	CIS	JP	JSP	OB	SCP	SIS	TR
CIS	0.864	0.917	0.786	**0.886**						
JP	0.883	0.919	0.740	0.551	**0.860**					
JSP	0.838	0.902	0.755	0.548	0.559	**0.869**				
OB	0.862	0.906	0.707	−0.478	−0.442	−0.448	**0.841**			
SCP	0.870	0.906	0.658	0.523	0.465	0.462	−0.667	**0.811**		
SIS	0.829	0.886	0.660	0.528	0.530	0.576	−0.537	0.573	**0.813**	
TR	0.824	0.895	0.740	0.513	0.595	0.451	−0.443	0.490	0.488	**0.860**

Diagonal bold numbers represent the square root of AVE.

**Table 3:** Cross-loadings.

Items	CIS	JP	JSP	OB	SCP	SIS	TR
CIS1	0.888						
CIS2	0.892						
CIS3	0.879						
JP1		0.862					
JP2		0.865					
JP3		0.859					
JP4		0.854					
JPS1			0.856				
JPS2			0.876				
JPS3			0.875				
OB1				0.845			
OB2				0.851			
OB3				0.854			
OB4				0.812			
SCP1					0.831		
SCP2					0.812		
SCP3					0.804		
SCP4					0.824		
SCP5					0.785		
SIS1						0.825	
SIS2						0.816	
SIS3						0.800	
SIS4						0.809	
TR1							0.881
TR2							0.864
TR3							0.835

We used the Fornell–Larcker criterion and heterotrait–monotrait ratio (HTMT) to test discriminant validity. As shown in Table 2, the square root of AVE was greater than the absolute value of the correlation coefficient of its row and column, which met the requirement (Fornell and Larcker, 1981). Moreover, Table 4 indicated that all HTMT values were lower than the threshold value of 0.85 (Kline, 2011). Therefore, the data had good discrimination validity.

**Table 4:** Heterotrait–Monotrait ratio (HTMT).

	CIS	JP	JSP	OB	SCP	SIS	TR
CIS							
JP	0.631						
JSP	0.644	0.647					
OB	0.552	0.504	0.524				
SCP	0.603	0.530	0.541	0.698			
SIS	0.624	0.620	0.692	0.632	0.675		
TR	0.609	0.696	0.542	0.523	0.578	0.591	

### 4.3. Hypothesis testing

We used the method of partial least squares structural equation modeling (PLS-SEM) and the SmartPLS 4.0 tool to test the structural equation model. PLS was chosen because it helps deal with high-order constructs and complex models, which is suitable for constructing and measuring new theories (Hair et al., 2019). This study tested social control (SC) as a second-order construct with three dimensions: TR, JP, and JSP.

**Figure 2:**
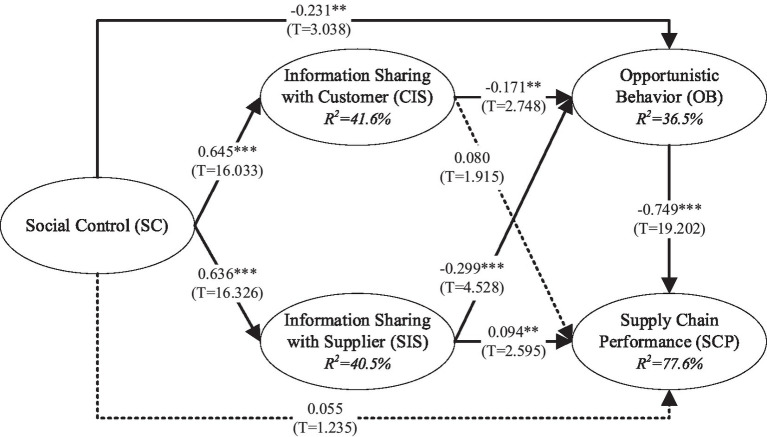
Hypothesis testing results. ^*^p < 0.05, ^**^p < 0.01, ^***^p < 0.001.

The cumulative explained variance variation of the SCP was 77.6%, and of the OB was 36.5%. As shown in Figure 2, SC has no influence on SCP (*β* = 0.055, *p* > 0.05) and has a negative effect on OB (*β* = −0.231, *p* < 0.01), indicating that H1 was not supported, and H2 was supported; SC positively impact on CIS (*β* = 0.645, *p* < 0.001) and SIS (*β* = 0.636, *p* < 0.001), supporting H3*a,b*; SIS has a positive influence on SCP (*β* = 0.094, *p* < 0.01), while CIS did not (*β* = 0.080, *p* > 0.05), thus, H4*a* was not supported and H4*b* was supported; both CIS (*β* = −0.171, *p* < 0.01) and SIS (*β* = −0.299, *p* < 0.001) have a negative influence on OB, supporting H5*a,b*. OB negatively influences SCP (*β* = −0.749, *p* < 0.001), supporting H6.

We also tested whether the control variables impact SCP. Results indicated that firm age (*β* = −0.021), firm size (*β* = 0.008), industries (*β* = 0.011), and position of subjects (*β* = −0.002) have no significant influence on SCP, and the cumulative explained variance variation of the SCP was only 1.4%.

### 4.4. *Post-hoc* analysis

To further deepen our understanding of the path of opportunistic behavior and performance improvement in the food supply chain from the perspective of social control, we tested the potential mediators using SmartPLS 4.0 tool in our research model. As shown in Table 5, information sharing with customers (*β* = −0.110, *t*-values = 2.625, *p* < 0.01) and information sharing with the supplier (*β* = −0.191, *t*-values = 4.460, *p* < 0.001) mediate the relationship between social control and opportunistic behavior; information sharing with the supplier (*β* = 0.060, *t*-values = 2.600, *p* < 0.01) and opportunistic behavior (*β* = 0.173, *t*-values = 3.047, *p* < 0.01) mediate the relationship between social control and supply chain performance; opportunistic behavior mediates the relationship between information sharing with the supplier (*β* = 0.224, *t*-values = 4.451, *p* < 0.001)/information sharing with the customer (*β* = 0.128, *t*-values = 2.747, *p* < 0.01) and supply chain performance. Moreover, the multiple mediation effects of information sharing with the customer and opportunistic behavior (*β* = 0.083, *t*-values = 2.626, *p* < 0.01)/information sharing with the supplier and opportunistic behavior (*β* = 0.143, *t*-values = 4.416, *p* < 0.001) between social control and supply chain performance were significant.

**Table 5:** Mediators testing results.

Paths	2.5%	97.5%	Beta (β)	*t*-value	*p* values
SC - > CIS - > OB	−0.202	−0.033	−0.110	2.625	0.009
SC - > CIS - > OB - > SCP	0.025	0.150	0.083	2.626	0.009
SC - > SIS - > OB	−0.276	−0.106	−0.191	4.460	0.000
SC - > SIS - > SCP	0.015	0.105	0.060	2.600	0.009
SC - > SIS - > OB - > SCP	0.080	0.207	0.143	4.416	0.000
SC - > OB - > SCP	0.059	0.282	0.173	3.047	0.002
SIS - > OB - > SCP	0.125	0.324	0.224	4.451	0.000
CIS - > OB - > SCP	0.040	0.224	0.128	2.747	0.006

## 5. Discussion

### 5.1. Key findings and theoretical implication

This study focuses on the opportunistic behavior of members in food supply chain management and the performance improvement of manufacturing enterprises. It introduces social control to investigate the direct path of social control’s influence on opportunistic behavior and supply chain performance and the indirect path of social control’s influence *via* information sharing as the mediating factor. In this research, the following conclusions are drawn.

First, social control can directly reduce the opportunistic behavior of food supply chain members. This conclusion is consistent with the view of Zhang et al. (2019) that social control is an important means to reduce the opportunism of partners. Furthermore, this research also finds that social control has multiple paths of indirectly restricting opportunistic behaviors. To be specific, social control can indirectly restrain opportunistic behaviors of supply chain partners *via* information sharing with customers and information sharing with suppliers. The mediating path can be represented as: SC → (+)CIS/SIS→(−)OB(*β* = −0.110, *p*<0.01/*β* = −0.191, *p*<0.001). Therefore, this study enriches the conclusions on the multiple influence paths of social control on the opportunistic behavior of supply chain members.

Furthermore, different from prior studies concluding that social control has a direct positive influence on supply chain performance (e.g., Li et al., 2010), this research finds that social control affects supply chain performance indirectly *via* various mediating factors instead of directly improving supply chain performance. Social control can reduce opportunistic behaviors *via* information sharing, thus enhancing supply chain performance. There are multiple mediating paths, that is, SC → (+)CIS/SIS→(−)OB → (−)SCP (*β* = 0.083, *p* < 0.01/*β* = 0.143, *p* < 0.001). Social control improves supply chain performance by weakening opportunism. So there exists a mediating path SC → (−)OB → (−)SCP (*β* = 0.172, *p* < 0.01). Thus, this research demonstrates the mediating effect of information sharing and opportunism in the relationship between social control and food supply chain performance.

Manufacturing enterprises’ information sharing with customers does not directly improve supply chain performance, whereas their information sharing with suppliers enhances supply chain performance. This conclusion is logical in the food supply chain scenario. Information sharing with suppliers can drive food manufacturing enterprises and their suppliers to make exchanges and cooperation concerning information on production, manufacturing, technology, raw materials, and other food production-related aspects. Thus, manufacturing enterprises can improve food processing technology and innovate in food production and circulation to guarantee their competitive advantage and food quality safety. However, information sharing with customers can only help manufacturing enterprises to understand better the market demand, timely track the market changes, and then gain supplementary information about food category development, thus facilitating the manufacturing enterprises in food design, taste improvement, and other fields, so information sharing with customers produces a limited influence on operating cost, food quality and delivery speed of the food supply chain. To further study the information-sharing mechanism, this research introduces a test of the path between information sharing with customers and information sharing with suppliers, and finds that information sharing with suppliers is not a mediating variable between information sharing with customers and supply chain performance improvement, and information sharing with customers and supply chain performance have a particular chain-linked mediating path: CIS → (+)SIS→(−)OB → (−)SCP (*β* = 0.045, *p* < 0.01). Specifically, information sharing with customers first affects information sharing with suppliers, acts on opportunistic behaviors, and eventually influences supply chain performance. This demonstrates paths of information sharing with customers to improve supply chain performance. Prior studies take information sharing as a concept (e.g., Huo et al., 2021), and investigate the effect of information sharing with suppliers on boosting the improvement of the overall supply chain quality (Ding et al., 2014; Christensen et al., 2021; Liu et al., 2021). This research deepens the understanding of information sharing in prior studies. It discusses the differences between information sharing with customers and information sharing with suppliers and differences in their influence mechanisms (that is, the influence mechanisms of information sharing with customers and information sharing with suppliers on supply chain performance and their correlations), thereby enriching the conceptual depth and conclusions of existing studies.

Lastly, this research concludes that both information sharing with customers and information sharing with suppliers can significantly reduce the opportunistic behavior of supply chain enterprises and thus enhance supply chain performance. This finding is inconsistent with Wang et al. (2014) that information sharing with suppliers does not correlate with opportunism.

In summary, social control can restrain opportunistic behavior and improve supply chain performance from the following four aspects. First, enterprises would incur severe losses from their “breach of trust.” Laws and regulations can adjust social behaviors, but trust, as the most critical psychological mechanism for effective communication in modern society, still takes an irreplaceable part, and can weaken the opportunistic motivation of upstream and downstream enterprises in the supply chain. Secondly, social control brings a special relationship between enterprises, so enterprise-enterprise relationships are no longer straightforward. Social control can drive upstream and downstream enterprises to make cooperation and daily communication on the premise of mutual benefits, so that the enterprises can be connected more closely, communicate with each other more smoothly, and run more efficiently, thus gaining more heterogeneous resources, cutting the communication cost, and significantly lowering the likelihood of opportunistic behavior triggered by information asymmetry. Thirdly, social control can establish relevant mechanisms and innovative working modes for manufacturing enterprises. Specifically, social control can help enterprises to build flexibly-enhancing, rights-equalizing, and resource-sharing mechanisms. These mechanisms can directly or indirectly enhance the operating efficiency and flexibility of the supply chain and strengthen the cooperation willingness and goal consistency between suppliers and manufacturers, thus significantly improving the supply chain performance. Lastly, social control can facilitate the fulfillment of cooperation between upstream and downstream enterprises in the supply chain. On this basis, design strategies and tactics, timely adjust the strategic orientation and industrial structure, lower losses and supply chain risks, and thus enhance the overall supply chain performance.

### 5.2. Policy suggestions

This research has great value for the practice of the food manufacturing industry to build and strengthen their relationships with supply chain partners and adopt the information-sharing mechanism to weaken opportunism and improve supply chain performance.

Food supply chain enterprises should pay attention to the supervision of opportunistic behavior, combat opportunism, and promote the food supply chain co-existence and co-prosperity. Especially in the context of the pandemic, people’s food preferences have changed dramatically, with consequent changes in food quotas and supplies for upstream farming, food processing and manufacturing, as well as suppliers and retailers. The pandemic increases the chances of opportunism. Based on our findings, a meaningful way to reduce or even avoid opportunism is to strengthen mutual trust and cooperation between enterprises, form an inter-organizational information sharing channel, and fix it as a common policy followed by member enterprises of the food supply chain.

We suggested that the food manufacturing industry build a social control mechanism based on the relationship of supply chain members. The traditional contract mechanism specifies the rights and obligations of supply chain enterprises. However, in the face of the outbreak of COVID-19, food supply chain enterprises are also faced with numerous difficulties, and relying solely on contracts may not be able to maintain the stability of the food supply chain. A single enterprise is likely to take opportunistic actions due to high profits or basic survival, resulting in the deactivation of the entire food supply chain. As found in this study, enhancing information sharing among supply chain firms through informal social control mechanisms to reduce opportunism and improve performance may be an essential way to increase the overall co-prosperity of the food supply chain in a pandemic environment. This also suggests that enterprises should, in addition to contracts, attach importance to strengthening mutual trust and cooperation with upstream and downstream enterprises to form a close cooperative relationship, deal with emergencies caused by the epidemic and achieve the overall prosperity of the supply chain and the performance of member enterprises.

We also suggested that the food manufacturing industry should consider the effect of information sharing to enhance supply chain management and cooperation. On the one hand, information-sharing platforms can be built so supply chain enterprises can share production, inventory, and transportation data. Enterprises at each node can effectively adjust their production plan for the current or next stage. On the other hand, it is feasible to invest more into the internal information construction of enterprises, strengthen the integration of internal departments of manufacturing enterprises, build new ways of information transmission to alter the original process, improve the flexibility of enterprises, and adopt social control to accelerate the response speed of the supply chain. Given the difference in the effect of information sharing with customers and information sharing with suppliers, manufacturing enterprises should share information strategically and selectively.

## 6. Conclusion and limitations

Ensuring enterprises’ sustainability and good performance is the inevitable choice in an uncertain environment (Wen et al., 2022). Especially for food supply chain enterprises, it is particularly challenging for them to reduce the harm of opportunistic behavior and maintain stable performance in the context of the pandemic. This study introduced social control and information sharing and built a model based on the OIPT theory. The study confirmed the positive effects of social control and information sharing on improving food supply chain performance and reducing opportunism. Therefore, this study provides a new perspective to explain the growth of supply chain performance and discovers the mediating mechanism of information sharing and the differences between the two types of information sharing. Unavoidably, this research has some limitations. Cross-sectional data were used in this research to show the actual running mechanism of the food supply chain. However, longitudinal tracking research may enrich research conclusions. Beyond that, existing research shows that social control and formal control complement each other (Li et al., 2010). Formal control can be applied to this research to discuss the similarities and differences between social control and formal control in their influence on opportunistic behavior and performance in the supply chain.

## Data availability statement

The raw data supporting the conclusions of this article will be made available by the authors, without undue reservation.

## Ethics statement

Ethical review and approval were not required for the study on human participants in accordance with the local legislation and institutional requirements. Written informed consent for participation was not required for this study in accordance with national legislation and institutional requirements. Written informed consent was obtained from the individual(s) for the publication of any potentially identifiable images or data included in this article.

## Author contributions

TL was responsible for idea generation, manuscript writing and revision. YG was responsible for hypothesis development and data analysis. QG was responsible for data collection. All authors contributed to the article and approved the submitted version.

## Funding

This study was supported by the Natural Science Foundation of Shandong Province of China (No. ZR2021QG007) and the Humanities and Social Science Fund of the Ministry of Education of China (No. 19YJC630118).

## Conflict of interest

The authors declare that the research was conducted in the absence of any commercial or financial relationships that could be construed as a potential conflict of interest.

## Publisher’s note

All claims expressed in this article are solely those of the authors and do not necessarily represent those of their affiliated organizations, or those of the publisher, the editors and the reviewers. Any product that may be evaluated in this article, or claim that may be made by its manufacturer, is not guaranteed or endorsed by the publisher.

## Appendix A. Measurement scales

**Social control** adapted from Claro et al. (2003) and Cai et al. (2009).

Joint planning

JP1 Our company plans food demands for the next seasons together with supply chain partners.
JP2 Our company plans the food types demands for the next seasons together with supply chain partners.
JP3 Our company tests market acceptance of new foods for the next seasons together with supply chain partners.
JP4 Our company shares long-term plans of food development for the next seasons together with supply chain partners.

Joint problem solving

JPS1 Our company and supply chain partners deal with problems that arise in the course of the relationship together.
JPS2 Our company and supply chain partners do not mind owing each other favors.
JPS3 Our company and supply chain partners share the responsibility for ensuring food quality and supply.

Trust

TR1 We expect supply chain partners to work with us for a long time.
TR2 Based on experience, we have complete confidence in relying on the supply chain partners to keep promises to us.
TR3 The selected supply chain partners are trustworthy.

**Information** Sharing adapted from Flynn et al. (2010) and Prajogo and Olhager (2012).

Information sharing with customer

CIS1 Our company shares marketing information with our primary customers.
CIS2 Our company keeps good communication with our primary customers.
CIS3 Our company keeps regular contact with major customers.

Information sharing with supplier

SIS1 Our major supplier participates in the food design and processing process.
SIS2 Our major supplier shares their production schedule with our company.
SIS3 We share our demand forecasts with our primary suppliers.
SIS4 We share our inventory levels with our primary suppliers.

**Supply Chain performance** adapted from Huo et al. (2017) and Prajogo and Olhager (2012).

SCP1 Our company can realize lower production costs than the competitors.
SCP2 Our company can achieve better food quality and stability than the competitors.
SCP3 Our company has an outstanding on-time delivery record with our customers.
SCP4 Our lead time for fulling customers’ personalized food needs is short.
SCP5 Our company can achieve a better food supply than the competitors*. (self-developed).

**Opportunistic behavior** adapted from Luo et al. (2015) and Huo et al. (2016a).

OB1 Our partners sometimes violate contractual terms and conditions.
OB2 Our partners increase their own gain by evading contractual obligations.
OB3 Our partners take advantage of contract leaks to expand their interests.
OB4 Our partners sometimes take advantage of contingencies to stop us from pursuing their responsibilities.
